# Ruxolitinib in steroid-refractory acute graft-vs-host disease: Japanese subgroup analysis of the randomized REACH2 trial

**DOI:** 10.1007/s12185-024-03772-6

**Published:** 2024-05-25

**Authors:** Takanori Teshima, Yasushi Onishi, Koji Kato, Shuichi Taniguchi, Koichi Miyamura, Kentaro Fukushima, Jun Kato, Takayuki Ishikawa, Noriko Doki, Hirohisa Nakamae, Yoshinobu Maeda, Yoshihiro Inamoto, Masaya Okada, Akio Maki, Fumika Shimada, Takeshi Tajima, Monika Wroclawska, Robert Zeiser, Makoto Onizuka

**Affiliations:** 1 Department of Hematology, Faculty of Medicine, Hokkaido University, Sapporo, Japan; 2 Department of Hematology, Tohoku University Hospital, Sendai, Japan; 3 Department of Hematology, Oncology and Cardiovascular Medicine, Kyushu University Hospital, Fukuoka, Japan; 4 Department of Hematology, Toranomon Hospital, Tokyo, Japan; 5 Department of Hematology, Japanese Red Cross Aichi Medical Center Nagoya Daiichi Hospital, Nagoya, Japan; 6 Department of Hematology and Oncology, Osaka University Graduate School of Medicine, Suita, Japan; 7 Division of Hematology, Department of Medicine, Keio University School of Medicine, Tokyo, Japan; 8 Department of Hematology, Kobe City Medical Center General Hospital, Kobe, Japan; 9 Hematology Division, Tokyo Metropolitan Cancer and Infectious Diseases Center, Komagome Hospital, Tokyo, Japan; 10 Graduate School of Medicine, Osaka City University, Osaka, Japan; 11 Department of Hematology, Oncology and Respiratory Medicine, Okayama University Graduate School of Medicine, Dentistry, and Pharmaceutical Sciences, Okayama, Japan; 12 Department of Hematopoietic Stem Cell Transplantation, National Cancer Center Hospital, Tokyo, Japan; 13 Hyogo College of Medicine, Kansai Medical University Medical Center, Hyogo, Japan; 14 Novartis Pharma K.K., Tokyo, Japan; 15 Novartis Pharma AG, Basel, Switzerland; 16 Department of Medicine I, Faculty of Medicine, Medical Center—Universi Medical Center - University of Freiburg, Freiburg, Germany; 17 Department of Hematology/Oncology, Tokai University School of Medicine, Kanagawa, Japan; 18 Present Address: Inuyama Chuo General Hospital, Inuyama, Japan

**Keywords:** Acute graft-versus-host disease, Ruxolitinib, JAK inhibitor, Japanese

## Abstract

Acute graft-versus-host disease (aGvHD) is a major complication after allogeneic hematopoietic stem cell transplantation in Japan and other countries. Nearly one-third of patients do not respond to standard systemic steroid therapy and no standard second-line treatment has been established in Japan. We report efficacy and safety findings of ruxolitinib versus best available therapy (BAT) from a subgroup analysis of the international, phase 3 REACH2 study in Japanese patients with steroid-refractory aGvHD. The primary endpoint was overall response rate (ORR) at day 28. Overall, 9 patients received ruxolitinib and 21 received BAT. The ORR at day 28 (88.9% vs 52.4%) and durable ORR at day 56 (66.7% vs 28.6%) were higher with ruxolitinib versus BAT. The estimated cumulative incidence of loss of response at 6 months was 12.5% with ruxolitinib and 18.2% with BAT. The median failure-free survival was longer with ruxolitinib versus BAT (2.73 vs 1.25 months). The most common adverse events up to day 28 in the ruxolitinib and BAT groups were anemia (55.6% vs 19.0%) and thrombocytopenia (44.4% vs 4.8%, respectively). Ruxolitinib showed better efficacy outcomes and a consistent safety profile compared with BAT in the Japanese subgroup, and the findings were consistent with overall study results.

**Supplementary Information:**

The online version contains supplementary material available at 10.1007/s12185-024-03772-6.

## Introduction

Hematopoietic stem cell transplantation (HSCT) has made groundbreaking progress by offering a potential curative treatment for a variety of hematological diseases. Similar to western countries, allogeneic HSCT is increasing in Japan, with annual numbers of almost 4000 in the recent years [1, 2]. Although the probability of survival after allogeneic HSCT has shown an improving trend in the last 10 years, the probability of survival at 1 year (64.2%) and at 5 years (48.5%) after HSCT is still not satisfactory [3], and one of the primary causes of non-relapse mortality (NRM) is graft-versus-host disease (GvHD), both in Japan and in other countries [4]. Systemic steroid therapy remains the standard first-line treatment for acute GvHD (aGvHD), although about one-third of patients do not respond to the therapy according to Japanese national registry data. Patients with steroid-refractory GvHD (SR-aGvHD) have poor prognosis, with a 2-year overall survival (OS) probability of 32.3% [5]. Secondary treatment options include antithymocyte globulin (ATG), extracorporeal photo-chemotherapy (ECP), mesenchymal stromal cell (MSC), mycophenolate mofetil (MMF), and TNF-α inhibitors (etanercept or infliximab); the majority of these are based on retrospective or non-randomized studies and ATG, MMF, and MSC had been approved in Japan for aGvHD [6, 7]. To date, there have been no head-to-head trials that showed superiority over other treatments, and SR-aGvHD remains with poor prognosis in Japanese patients.

Janus kinase (JAK) signaling pathways play a role in regulating the development, proliferation, and activation of several immune cell types important for GvHD pathogenesis, including dendritic cells, macrophages, T cells, B cells, and neutrophils via a variety of cytokines signaling [8, 9]. Ruxolitinib is an orally administered selective JAK1/2 inhibitor approved in treatment of adult and pediatric patients with SR-aGvHD or cGvHD by the FDA and with aGvHD or cGvHD by the European Commission. [10–12]. REACH2 is a randomized, phase 3, international, open-label study conducted to investigate the efficacy and safety of ruxolitinib compared with best available therapy (BAT) added to the patient’s immunosuppressive regimen. This study met the primary endpoint with a significantly higher overall response rate (ORR) at day 28 (62.3% vs 39.4%; odds ratio, 2.64; 95% CI, 1.65–4.22; *P* < 0.001), and its key secondary endpoint with a higher durable overall response at day 56 (39.6% vs 21.9%; odds ratio, 2.38; 95% CI, 1.43–3.94; *P* < 0.001) of ruxolitinib treatment when compared with BAT among the patients with grade II–IV SR aGvHD [13, 14]. This manuscript presents a subgroup analysis of 30 Japanese patients enrolled in the REACH2 study with a minimum follow-up of 6 months or earlier discontinuation.

## Materials and methods

### Study design

The eligibility criteria and study design of the REACH2 study have been described previously [13]. Briefly, REACH2 (ClinicalTrials.gov number: NCT02913261) is a multicenter, open-label, randomized, international phase 3 study that evaluated the efficacy and safety of ruxolitinib compared with investigator’s choice of BAT in 309 patients with SR-aGvHD. Eligible patients were randomized (1:1) to receive either ruxolitinib (10 mg twice daily) or BAT for up to 24 weeks. Randomized patients were stratified by aGvHD grade (II vs. III vs. IV) randomization. Tapering of ruxolitinib dose was allowed after day 56 in patients who had a response and after discontinuation of corticosteroids. Crossover to ruxolitinib from BAT was allowed after day 28 in patients who had no response at day 28 (failed to meet the primary endpoint definition) or had loss of response thereafter and received additional systemic therapy and had no signs of chronic GvHD (cGvHD). Investigator-selected BAT options included ATG, ECP, MSCs, methotrexate, MMF, mammalian target of rapamycin (mTOR) inhibitor (everolimus or sirolimus), etanercept, or infliximab. The data cutoff date for the primary analysis was July 25, 2019 and the secondary endpoints reported here are based on the data cutoff at January 6, 2020 (minimum follow-up of 6 months).

Alongside the continued use of calcineurin inhibitors (CNI; cyclosporine or tacrolimus) and glucocorticoids, standard supportive therapy was allowed in both the treatment groups. Other prior systemic immunosuppressive treatments had to be discontinued unless used for aGvHD prophylaxis (i.e., started before the diagnosis of aGvHD). Patient visits were scheduled weekly from day 1 to day 56 followed by every 4 weeks up to week 24, unless a prolonged tapering period was necessitated. At 30 days after the last dose of trial treatment, a safety follow-up visit was scheduled and long-term follow-up visits were scheduled at months 6, 9, 12, 18, and 24 post-randomizations to collect data on survival, progression, and safety outcomes.

Patients who crossed over to ruxolitinib were followed until completion of treatment and received the same treatment and tapering schedule as patients randomized to ruxolitinib treatment.

Randomization (1:1) was implemented for the global population and not stratified by geography, therefore the patient number for the Japanese subgroup may not exactly reflect the 1:1 allocation.

The trial was designed and conducted in accordance with the guidelines for Good Clinical Practice of the International Council for Harmonization, with applicable local regulations, and with the principles of the Declaration of Helsinki. The protocol was approved by the relevant institutional review board, independent ethics committee, or research ethics board at each participating center. Signed informed consent was obtained from the participating patient, parent, or guardian.

### Patient population

The study included adults and adolescents (aged ≥ 12 years) with grade II–IV SR-aGvHD who underwent allogeneic stem cell transplantation (alloSCT) and had evidence of myeloid and platelet engraftment (absolute neutrophil count [ANC] > 1000/mm^3^ and platelet count > 20,000/mm^3^).

Patients were excluded if they had received more than 1 prior treatment for SR-aGvHD; had failed prior alloSCT in the previous 6 months; had a relapsed primary cancer after undergoing alloSCT; had received JAK inhibitor therapy for any indication after the initiation of alloSCT conditioning; or had an active, uncontrolled infection.

### Study endpoints

The primary endpoint was ORR at day 28 (according to Harris’s criteria [15], defined as the proportion of patients who had a complete response or partial response as compared with baseline organ staging without the use of additional systemic therapy for aGvHD). The day 28 time point was chosen for ORR based on earlier reports that showed better correlation with subsequent long-term survival [5]. The key secondary endpoint was durable overall response at day 56 (defined as the proportion of patients in each treatment group who had a response at day 28 that was maintained up to day 56).

Other secondary endpoints included duration of response, best overall response, failure-free survival (FFS), NRM, incidence of malignancy relapse/progression (MR), and incidence of cGvHD. Duration of response was defined as the time from first response to aGvHD progression or the addition of new systemic therapy for aGvHD; competing risks were the onset of cGvHD or death without progression of aGvHD. Best overall response was defined as the proportion of patients with a complete or partial response at any time up to and including day 28 and before the start of additional systemic therapy for aGvHD. Failure-free survival was defined as time from randomization to relapse or progression of hematologic disease, non–relapse-related death, or the addition of new systemic therapy for aGvHD; the competing risk was the onset of cGvHD. Non-relapse mortality was defined as the time from randomization to death not preceded by hematologic disease relapse or progression; the competing event was relapse or progression of hematologic disease. Malignancy relapse/progression was defined as time from randomization to relapse or progression of hematologic cancer; the competing risk was non–relapse-related death.

Safety was assessed by monitoring the frequency, duration, and severity of adverse events (AEs) and serious AEs (SAEs), including the occurrence of any infection or second primary cancer, by means of routine physical examination and laboratory assessments; AEs were graded according to the Common Terminology Criteria for Adverse Events, version 4.03.

Pharmacokinetics (PK) were assessed by collecting plasma samples. Serial blood samplings were performed at predose and 0.5, 1.0, 1.5, 2, 4, 6, and 9 h postdose on day 1 and day 7 for a subgroup of patients to calculate PK parameters in plasma with a non-compartmental method using Phoenix WinNonlin® (Pharsight, Mountain View, CA), maximum plasma concentration (C_max_), time to reach the maximum concentration (T_max_), and area under the plasma concentration–time curve to the last measurable concentration (AUC_last_). Sparse samplings including trough concentrations were conducted in all patients during the study. Plasma concentrations were measured using a validated liquid chromatography-tandem mass spectrometry method with the lower limit of quantification at 0.500 ng/mL.

### Statistical analysis

In the REACH2 study, a target of 308 patients expected to have 90% power to test the primary endpoint (ORR at day 28) and approximately 90% power to test the secondary endpoint (durable ORR at day 56). The full analysis set included all patients who underwent randomization and the safety analysis set included all patients who received at least 1 dose of trial treatment. The PK analysis set (PAS) included all patients who provided at least one evaluable PK concentration. The PAS was used for all PK data analysis. Japanese patients enrolled in the global REACH2 study were summarized primarily in a descriptive manner in this report due to limited numbers. The primary and key secondary endpoints were presented according to randomized treatment group with a two-sided exact binomial 95% confidence interval. Cumulative-incidence curves were estimated for FFS, duration of response, NRM, MR, and incidence of cGvHD for each treatment group. Kaplan–Meier curves for FFS were plotted and the hazard ratios were calculated, along with the 95% confidence intervals, with the use of a stratified Cox model.

## Results

### Patient characteristics

**Fig. 1:**
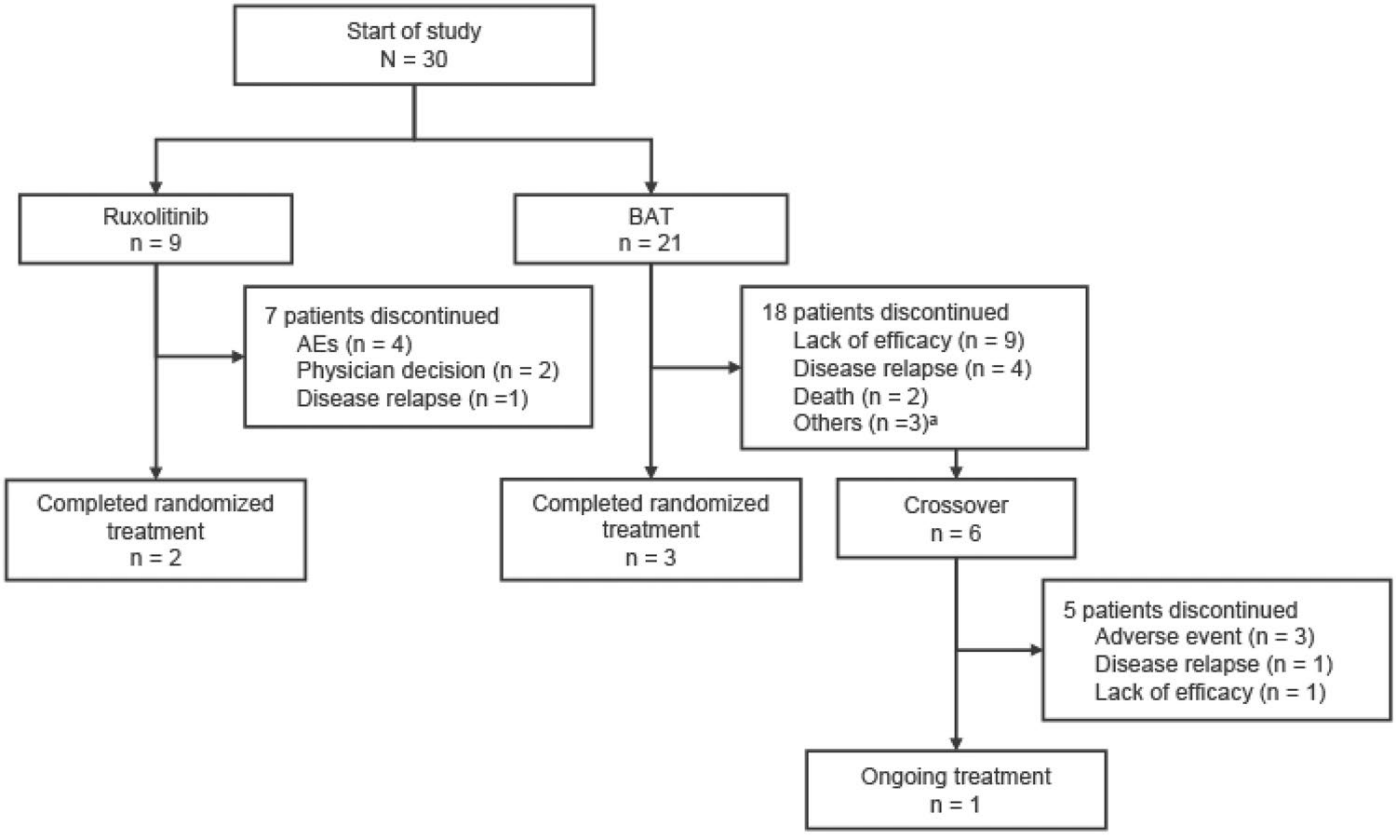
Patient disposition in the Japanese subgroup. ^a^Includes treatment discontinuation due to physician decision (n = 1), failure to meet protocol continuation criteria (n = 1), or subject/guardian decision (n = 1). AE, adverse event; BAT, best available therapy

Thirty Japanese patients were randomized to receive either ruxolitinib or BAT (9 and 21 patients, respectively) as shown in Fig. 1. The most common initial BAT selected in Japan was MSC, received by 11 of 21 patients (52.4%), followed by ATG (6 patients, 28.6%). Four patients received at least 2 BAT medications. At data cutoff, 2 patients in the ruxolitinib group and 3 patients in the BAT group completed the study treatment. Of the 21 patients in the BAT group, 6 (28.6%) crossed over to receive ruxolitinib on or after day 28. Four patients in the ruxolitinib group and 9 in the BAT group entered the long-term follow-up.

Among the randomized Japanese patients, median age was 57.5 years (range, 18–69), grade II and III aGvHD was noted in 50% of patients for each grade, and the distribution of patients according to aGvHD severity grade was similar (Table 1). Both baseline and demographic characteristics were similar between the treatment groups. The median time from diagnosis of grade ≥ II aGvHD to randomization was 29 days (range: 7–171 days) in the ruxolitinib group and 23 days (range: 3–129 days) in the BAT group.

**Table 1:** Demographics and baseline characteristics

Characteristics	Ruxolitinib (n = 9)	BAT (n = 21)	Total (N = 30)
Age			
Median (range), years	58.0 (21–68)	57.0 (18–69)	57.5 (18–69)
18–65 years, n (%)^a^	8 (88.9)	20 (95.2)	28 (93.3)
> 65 years, n (%)	1 (11.1)	1 (4.8)	2 (6.7)
Steroid-refractory criteria, n (%)			
Progression after at least 3 days	2 (22.2)	4 (19.0)	6 (20.0)
Failure to respond after 7 days	3 (33.3)	5 (23.8)	8 (26.7)
Failure during steroid taper	4 (44.4)	12 (57.1)	16 (53.3)
Overall aGvHD grade at baseline^a^, n (%)			
Grade II	4 (44.4)	11 (52.4)	15 (50.0)
Grade III	5 (55.6)	10 (47.6)	15 (50.0)
aGvHD organ involvement, n (%)			
Skin	4 (44.4)	6 (28.6)	10 (33.3)
Liver	0	3 (14.3)	3 (10.0)
Upper GI	2 (22.2)	6 (28.6)	8 (26.7)
Lower GI	8 (88.9)	17 (81.0)	25 (83.3)
Stem cell type, n (%)			
Bone marrow	5 (55.6)	7 (33.3)	12 (40.0)
Peripheral blood	3 (33.3)	9 (42.9)	12 (40.0)
Single cord blood	1 (11.1)	5 (23.8)	6 (20.0)
Conditioning regimen type, n (%)			
Myeloablative	4 (44.4)	9 (42.9)	13 (43.3)
Non-myeloablative	3 (33.3)	5 (23.8)	8 (26.7)
Reduced intensity	2 (22.2)	7 (33.3)	9 (30.0)
HLA match score, n (%)			
Matched			
10/10	0	1 (4.8)	1 (3.3)
8/8	4 (44.4)	5 (23.8)	9 (30.0)
6/6	1 (11.1)	0	1 (3.3)
Mismatched			
7/8	2 (22.2)	5 (23.8)	7 (23.3)
6/8	1 (11.1)	3 (14.3)	4 (13.3)
5/8	1 (11.1)	4 (19.0)	5 (16.7)
4/8	0	3 (14.3)	3 (10.0)
Donor type (source of grafts), n (%)			
Not related	6 (66.7)	16 (76.2)	22 (73.3)
Related	3 (33.3)	5 (23.8)	8 (26.7)
Prior aGvHD therapy, n (%)			
Steroid + CNI	6 (66.7)	8 (38.1)	14 (46.7)
Steroid + CNI + other systemic aGvHD treatment	3 (33.3)	13 (61.9)	16 (53.3)
Steroid + CNI + only aGvHD prophylaxis	0	5 (23.8)	5 (16.7)
Steroid + CNI + only aGvHD treatment	1 (11.1)	3 (14.3)	4 (13.3)
Steroid + CNI + both aGvHD prophylaxis and treatment	2 (22.2)	5 (23.8)	7 (23.3)
Initial BAT therapy	–		–
Mesenchymal stromal cells		11 (52.4)	
Anti-thymocyte globulin		6 (28.6)	
Infliximab		2 (9.5)	
Mycophenolate mofetil		2 (9.5)	

*aGvHD* acute graft-versus-host disease, *BAT* best available therapy, *CNI* calcineurin inhibitor, *GI* gastrointestinal, *HLA* human leukocyte antigen

^a^The overall study eligibility was ≥ 12 years age, however there were no patients enrolled below 18 years in the Japanese subgroup

The underling diseases, malignant in all patients, primarily included myelodysplastic syndrome (MDS; 22.2%, 42.9%) and acute myeloid leukemia (AML; 55.6%, 23.8%) in the ruxolitinib vs BAT groups, respectively (Table S1). The stem cell sources were bone marrow (55.6%, 33.3%), peripheral blood (33.3%, 42.9%), and single umbilical cord blood (11.1%, 23.8%) in the ruxolitinib vs BAT groups, respectively (Table 1). The proportion of HSCT from human leukocyte antigen (HLA)-matched donors was 55.6% in the ruxolitinib group and 28.6% in the BAT group.

The median duration from the diagnosis of grade II–IV aGvHD to the diagnosis of SR-aGvHD was 27 days in the ruxolitinib group and 13 days in the BAT group. In both groups, the most common reason for the diagnosis of SR-aGvHD was “failure during corticosteroid taper” (44.4% vs 57.1%). The most common aGvHD organ involvement in the Japanese population was lower gastrointestinal tract (GI) in both groups (88.9%, 81.0%).

Prior aGvHD treatment was a combination of steroid and CNI in 66.7% vs 38.1% patients, and a combination of steroid, CNI, and other systemic aGvHD treatment in 33.3% vs 61.9% patients in ruxolitinib vs BAT groups, respectively.

### Pharmacokinetics (PK)

PK parameters were obtained in an adult Japanese patient in the ruxolitinib group on each sampling day of day 1 and day 7. The individual values of C_max,_ T_max,_ and AUC_last_ were 99.9 ng/mL, 2.13 h, and 398 ng∙h/mL on day 1, and 89.0 ng/mL, 2.00 h, and 599 ng∙h/mL on day 7, respectively. Trough concentrations in Japanese adult patients in the ruxolitinib group were obtained postdose up to week 24. The individual data ranged from 0.801–106 ng/mL (n = 1–7). The Japanese data of PK parameters and trough concentrations were almost within the range of individual values in non-Japanese patients.

### Efficacy

**Fig. 2:**
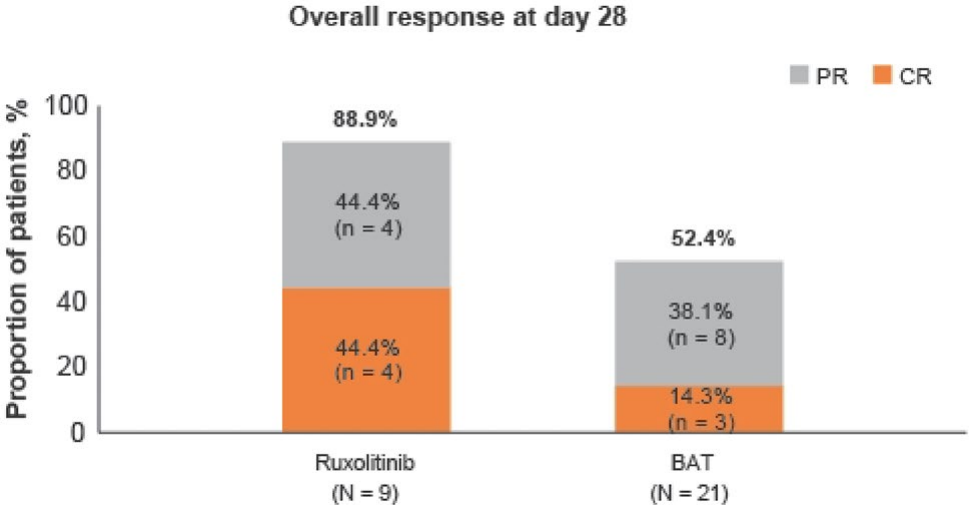
Overall response at day 28. CR, complete response; ORR, overall response rate; PR, partial response

**Fig. 3:**
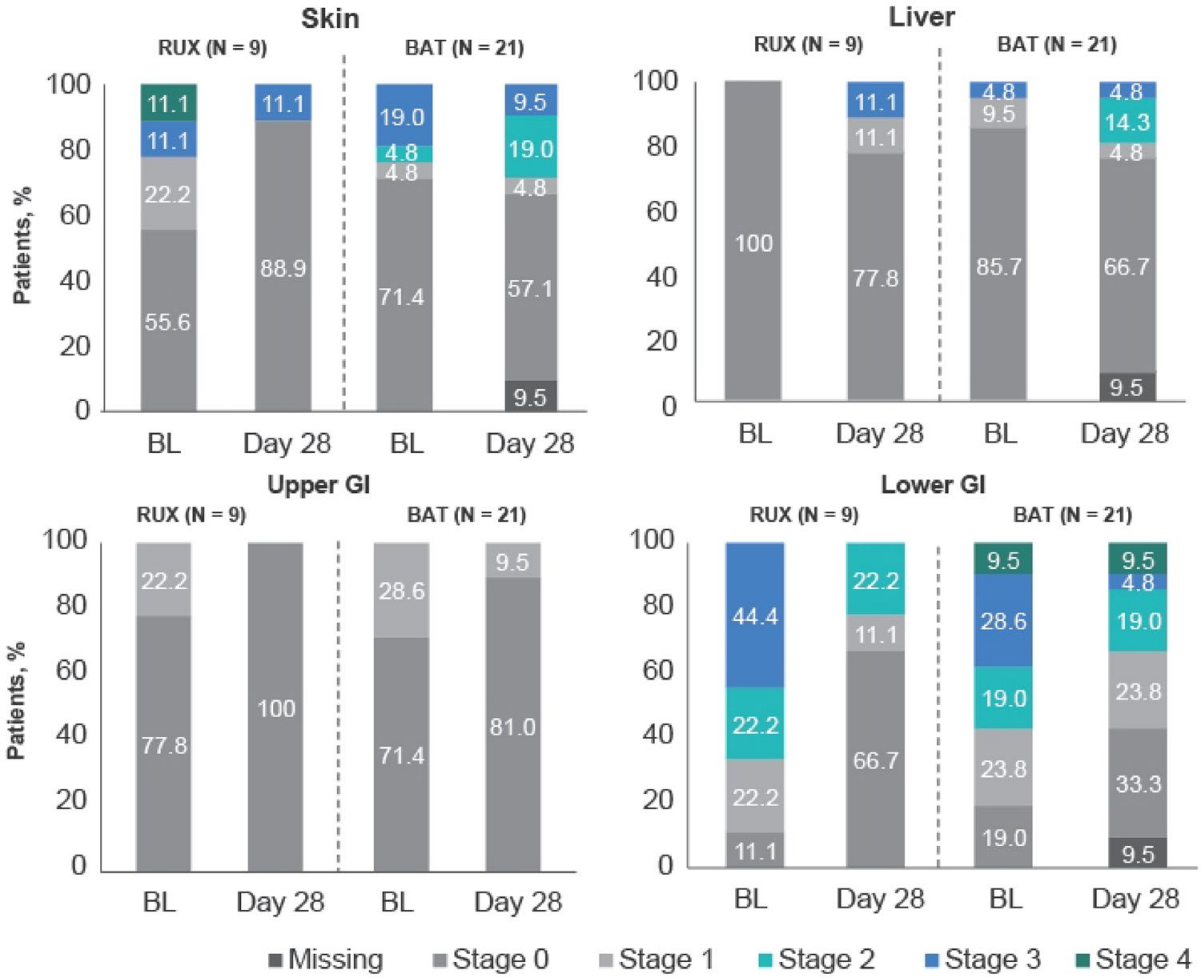
Shift in aGvHD organ staging from baseline to day 28. BL, baseline; GI, gastrointestinal

The ORR at day 28, the primary endpoint, was higher in the ruxolitinib group than in the BAT group [88.9% (8 of 9 patients) vs 52.4% (11 of 21 patients); odds ratio, 7.0; 95% confidence interval (CI), 0.81–60.4] (Fig. 2). The percentage of patients with a complete response was 44.4% (4 patients) and 14.3% (3 patients), respectively. The number of responders in the BAT group was 9 patients for MSC and 1 each for ATG and MMF. Improvement in the aGvHD grade for skin, liver, upper GI, and lower GI involvement at day 28 is shown for each treatment group in Fig. 3. Improvement by organ stage was noted in both the groups and no worsening was reported in the ruxolitinib group, except for liver involvement in 2 patients (Table S2). Lower GI was the most common organ involved in Japanese patients, including 8 patients with organ stage 3 (n = 4), stage 2 (n = 2), and stage 1 (n = 2) at baseline in the ruxolitinib group. Seven patients showed improvement of lower GI GvHD stage and 1 patient showed no change at day 28. In comparison, 8 out of 17 patients showed lower GI improvement in the BAT group.

Six patients in BAT crossed over to ruxolitinib, 3 of whom had achieved complete response at crossover day 28.

**Fig. 4:**
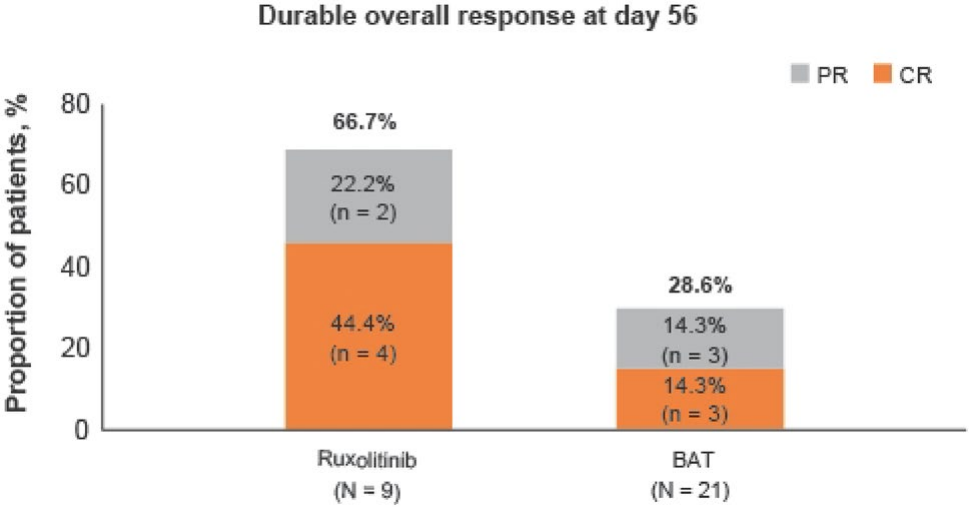
Durable overall response at day 56. CR, complete response; ORR, overall response rate; PR, partial response. Durable overall response was defined as the proportion of patients in each treatment group who had a response at day 28 that was maintained up to day 56

Durable overall response at day 56 was higher in the ruxolitinib group than in the BAT group [66.7% (6 patients) vs 28.6% (6 patients); odds ratio, 6.08; 95% CI, 0.88–42.1; Fig. 4]. In the ruxolitinib group, 6 responders at day 28 maintained the response until day 56.

The best overall response at day 28 (percentage of patients who had a complete or partial response at any time up to and including day 28 and before the start of additional systemic therapy for aGvHD) was 100% (9 patients) in the ruxolitinib group and 66.7% (14 patients) in the BAT group. The estimated cumulative incidence of loss of response at 6 months was 12.5% (95% CI, 0.46–44.82) in the ruxolitinib group and 18.2% (95% CI, 2.47–45.6) in the BAT group.

**Fig. 5:**
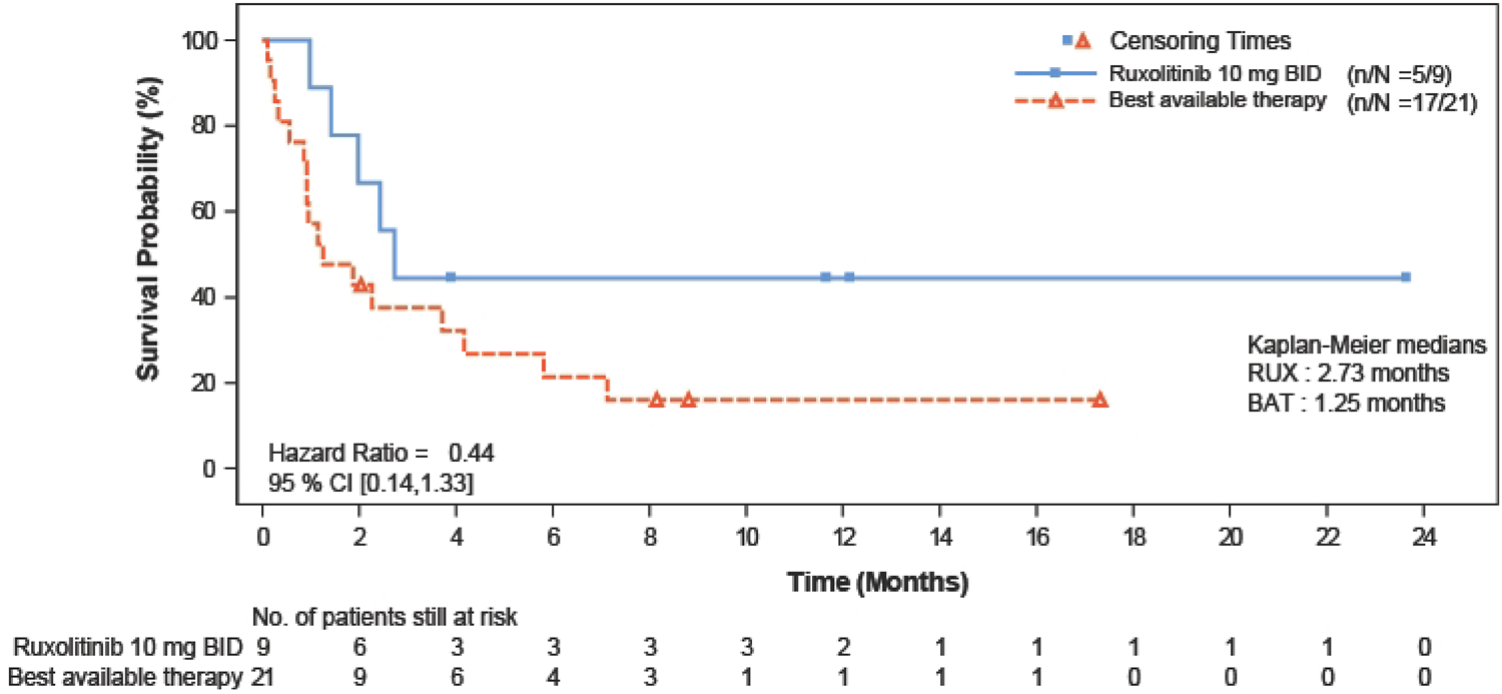
Kaplan–Meier plot of failure-free survival (FAS). BID, twice daily; CI, confidence interval; FAS, full analysis set

**Fig. 6:**
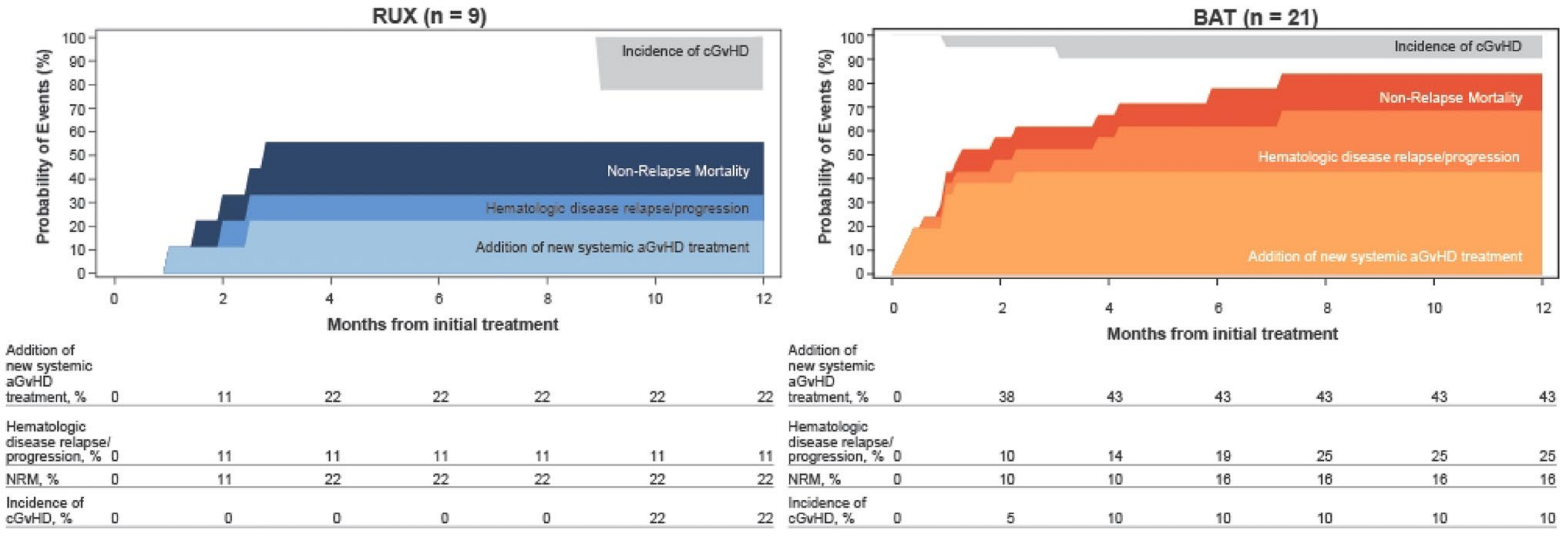
Failure-free survival by treatment (FAS). aGvHD, acute graft-versus-host disease; cGvHD, chronic graft-versus-host disease; FAS, full analysis set; NRM, non-relapse mortality

The median FFS was 2.73 months in the ruxolitinib group and 1.25 months in the BAT group (hazard ratio, 0.44; 95% CI, 0.14–1.33) (Fig. 5). The estimated cumulative incidence of events (earliest event of NRM, hematologic disease relapse/progression, or addition of new systemic aGvHD treatment) at 1 month was lower in the ruxolitinib group than in the BAT group (11.1% vs. 42.9%) and remained lower at all the time points up to 12 months (55.6% vs NE; Fig. 6).

Up to data cutoff, underlying malignancy relapse or progression occurred in 1 and 6 patients in the ruxolitinib and BAT groups, respectively. The cumulative incidence of cancer relapse or progression at 12 months was 11% (95% CI 0.4–41.2) in the ruxolitinib group and 30% (11.5–51.3) in the BAT group; NRM occurred in 4 patients in the ruxolitinib group and 10 patients in the BAT group. Cumulative incidence of NRM was lower with ruxolitinib than BAT at month 1 (0 vs 4.8%) and remained stable until 12 months (44.4% vs 52.9%).

One patient receiving ruxolitinib and 4 patients receiving BAT experienced cGvHD. Overall severity of cGvHD was mild in 1 patient in the ruxolitinib group and 3 patients in the BAT group, whereas 1 patient in BAT experienced moderate severity.

### Safety

Treatment was discontinued in 7 of 9 patients (77.8%) in the ruxolitinib group and in 18 of 21 (85.7%) in the BAT group. The most common reason was AEs in ruxolitinib [4 (44%)] and lack of efficacy in BAT (9 [43%]). The median duration of exposure to therapy was 72 days (range, 14–335) in the ruxolitinib group and 29 days (range, 1–68) in the BAT group. The median dose intensity of ruxolitinib was 11.4 mg per day (interquartile range, 8.8–15.0) and it was slightly lower than the overall population (16.6 mg) [13]. The median duration of exposure to ruxolitinib was comparable between the Japanese patients [72 days (range, 14–335)] and the overall REACH2 population [63 days (range, 6–463)].

The most common AEs (of any grade and of grade ≥ 3) in Japanese patients were anemia, thrombocytopenia, and decrease in platelet and white blood cell count (Table 2). Serious AEs were reported in 6 patients (grade ≥ 3, 66.7%) in the ruxolitinib group and 10 patients (grade ≥ 3, 42.9%) in the BAT group, and the most common event was sepsis and acute kidney injury in the ruxolitinib and BAT groups (n = 2, each) respectively.

**Table 2:** Most frequent adverse events up to day 28 (occurring in ≥ 10% patients in any group; safety set)

Event	Ruxolitinib, N = 9		BAT, N = 21	
	Any grade, n (%)	Grade ≥ 3, n (%)	Any grade, n (%)	Grade ≥ 3, n (%)
Any adverse event	9 (100)	8 (88.9)	19 (90.5)	16 (76.2)
Anemia	5 (55.6)	4 (44.4)	4 (19.0)	4 (19.0)
Thrombocytopenia	4 (44.4)	3 (33.3)	1 (4.8)	0
Platelet count decreased	4 (44.4)	4 (44.4)	5 (23.8)	5 (23.8)
White blood cell count decreased	3 (33.3)	3 (33.3)	4 (19.0)	4 (19.0)
Nausea	3 (33.3)	0	0	0
Vomiting	2 (22.2)	0	0	0
Edema peripheral	2 (22.2)	0	2 (9.5)	0
Cytomegalovirus infection	2 (22.2)	1 (11.1)	2 (9.5)	2 (9.5)
Alanine aminotransferase increased	2 (22.2)	1 (11.1)	1 (4.8)	1 (4.8)
Blood creatinine increased	2 (22.2)	0	2 (9.5)	0
Acute kidney injury	2 (22.2)	0	1 (4.8)	1 (4.8)
Hypoalbuminemia	1 (11.1)	1 (11.1)	4 (19.0)	2 (9.5)
Hypokalemia	1 (11.1)	0	5 (23.8)	1 (4.8)

*BAT* best available therapy

Up to data cutoff, AEs led to dose modifications in 6 patients (89%) who had received ruxolitinib and 2 patients (9.5%) who had received BAT, and to treatment discontinuation in 4 (44.4%) and none, respectively. Common AEs leading to dose modification in ruxolitinib were decreased platelet count (n = 4), abnormal hepatic function (n = 2), and increased blood creatinine levels (n = 2). Sepsis (n = 2) was the most common AE leading to ruxolitinib discontinuation.

Infections up to data cutoff occurred in 7 patients (78%) receiving ruxolitinib and 17 patients (81%) receiving BAT (Table 3). Viral and bacterial infections were the most common type of infection in both treatment groups. Grade 3 infections by infection severity grade reported in ruxolitinib were 3 fungal infections (candida infection, n = 2; bronchopulmonary aspergillosis, n = 1), 1 viral infection (cytomegalovirus, n = 1), and 3 bacterial infections (enterococcal infection, n = 1; sepsis, n = 2). Malignancies did not occur in the Japanese population.

**Table 3:** Infections (grade ≥ 3 events occurring in ≥ 10% of patients in any group; safety set)

Infection	Ruxolitinib, N = 9	BAT, N = 21
Maximum severity grade		
Number of subjects with at least one event, n (%)	7 (77.8)	17 (81.0)
Grade 1	0	3 (14.3)
Grade 2	2 (22.2)	11 (52.4)
Grade 3	5 (55.6)	3 (14.3)
Type of Infection, n (%)		
Fungal infection	4 (44.4)	2 (9.5)
Candida infection (grade 3)	2 (22.2)	–
Bronchopulmonary aspergillosis (grade 3)	1 (11.1)	–
Viral infection	5 (55.6)	12 (57.1)
CMV infection (grade 3)	1 (11.1)	–
Bacterial infection	5 (55.6)	11 (52.4)
Sepsis (grade 3)	2 (22.2)	–
Enterococcal infection (grade 3)	1 (11.1)	–
Unknown	1 (11.1)	2 (9.5)

*BAT* best available therapy, *CMV* cytomegalovirus

A total of 5 patients (56%) in the ruxolitinib group and 15 patients (71%) in the BAT group had died by the data cutoff (median duration of randomized treatment period, 83 days vs 58 days). Two deaths in the ruxolitinib group and 4 in the BAT group were attributed to aGvHD. The other causes of death in the ruxolitinib group included sepsis, AML, and renal impairment (1 patient each).

## Discussion

Steroid refractory aGvHD is a major complication after allogeneic HSCT that can cause morbidity and mortality [16]. Although occurrence of aGvHD in Japan is less common than in western countries, patients who do not respond to initial steroid therapy have poor prognosis [17]. In Japan, only MMF, ATG and MSC have been approved for treatment-refractory aGVHD [7, 18–20]. Currently, there is no standard second-line therapy established for aGvHD worldwide. [13, 21]. This may be attributed to data coming mainly from retrospective, single-arm, phase 2 studies and not randomized trials, which makes it difficult to establish superiority of 1 therapy over another [16]. Since REACH2 is an international, prospective, randomized phase 3 trial, the outcomes may support the standardization of therapy options for SR-aGvHD [13]. However, 6-month follow-up safety and efficacy results of REACH2 had demonstrated sustained advantage of ruxolitinib over BAT; no new safety signals were observed in longer exposure to ruxolitinib [22]. Although this subgroup analysis was limited due to the small number of patients and the variability of data and need to be carefully interpreted, Japanese population showed clinically significant efficacy and safety as the whole study population.

REACH2 is the first randomized trial on SR-aGvHD that showed superiority to BAT. The BAT chosen by Japanese investigators reflected current available therapies in Japan and were predominantly MSC and ATG. The most common initial BAT was MSC, received by 11 of 21 patients (52.4%), followed by ATG (6 patients, 28.6%). In the BAT group, the number of responders was 9 patients for MSC and one each for ATG and MMF at day 28. Its response was almost PR and 6 of 11 patients (54%) achieved durable response (CR + PR) at day 56 while 6 of 8 patients (75%) in the ruxolitinib group achieved durable response at day 56. Although it tended to be different from the overall study where ECP and MMF were more frequently used, higher ORR at day 28 (88.9% vs 52.4%) in ruxolitinib vs BAT was seen in the Japanese population and the response was durable up to day 56 (66.7% vs 28.6%). A longer FFS in ruxolitinib compared with the BAT group was also seen in the Japanese population. The best overall response was higher with ruxolitinib in the Japanese subgroup (100%) than in the overall population (82%).

Zeiser et al. previously reported that ruxolitinib was effective irrespective of GvHD organ involvement at baseline in the REACH2 global study analysis including Japanese patients [23]. In this report, although patient numbers were limited, the majority of the Japanese patients showed improvement in organ stages for each of the organs except liver GvHD. In the Japanese population, there were no patients with liver GvHD who were randomized to ruxolitinib, and lower GI was the major organ involved in this subpopulation. The majority of patients with lower GI-GvHD (7/8) and all with upper-GI GvHD (2/2) showed improvement in organ stage at day 28 compared to baseline, suggesting that oral treatment with ruxolitinib can also be used effectively in patients with GI-GvHD.

At the data cutoff, 2 patients were completely tapered off from ruxolitinib treatment. In the REACH2 study, ruxolitinib tapering was allowed after day 56 and after steroids were discontinued. Based on the predefined tapering guidance provided in the protocol, investigators were allowed to taper off the ruxolitinib treatment based on evaluation of the patient’s condition, current dosing regimen, and the clinical judgement of the investigator. The tapering guidance was 50% dose reduction every 2 months (56 days), i.e., initial dose reduction from 10 mg twice daily to 5 mg twice daily and, if sustained aGvHD stable disease is observed, the patient is further tapered by a second 50% dosage reduction to 5 mg orally once daily for an additional 56 days, prior to cessation.

The safety profile of ruxolitinib in the Japanese subgroup was consistent with the overall REACH2 study safety findings and the known safety profile of ruxolitinib, and as expected in patients with SR-aGvHD [13, 24, 25]. Ruxolitinib dose modifications were needed in about 89% of patients and 44.4% of patients discontinued ruxolitinib owing to AEs. In this study, ruxolitinib dose adjustments were mandatory for patients who had treatment-related grade 3/4 neutropenia and grade 4 thrombocytopenia. No recommendations for BAT dose adjustments were defined in the protocol and these were adjusted as per standard of care. Cytopenias, predominantly thrombocytopenia and anemia, were the most common AEs reported with ruxolitinib in the study. Most cytopenia events were manageable with ruxolitinib dose adjustments or interruption, and events leading to treatment discontinuation were few. Dose intensity in the Japanese was slightly lower than in the whole study population, but dose exposure was slightly longer than the REACH2 overall population, suggesting that ruxolitinib treatments were well maintained by dose adjustment in Japanese patients. The incidence of infections was similar in both the Japanese and the overall population, and between both the treatment groups. The incidence of cytomegalovirus infection was in line with those reported in the overall population for the ruxolitinib group [13].

The deaths and SAEs reported in the Japanese patients receiving ruxolitinib reflected the characteristics of infections expected in immunocompromised patients under immunosuppression, the underlying disease, events expected after HSCT or the serious disease under study but were not unique to the Japanese patients. There was no substantial difference in the incidence of deaths between the two groups, which was in line with the overall population and previously reported findings with ruxolitinib in patients with SR-aGvHD.

Pharmacokinetics in adolescent and adult patients were assessed in the study. There were no apparent differences in PK between adolescent and adult patients (data not presented). The PK parameters and trough concentrations were obtained in adult Japanese patients and the results were almost within the range in non-Japanese patients, suggesting PK ethnic insensitivity consistent with the results in healthy volunteers and patients in the other indications (myelofibrosis and polycythemia vera). Furthermore, although the majority of the Japanese patients had lower GI-GvHD, including nearly half of those with stage 3, ruxolitinib was absorbed and exerted efficacy, as discussed above.

In conclusion, ruxolitinib therapy led to a numerically higher overall response than BAT at day 28 and a higher durable overall response at day 56 among patients with grade II–IV SR-aGvHD. With ruxolitinib, there was a higher incidence of thrombocytopenia and a modestly higher incidence of anemia, while the infection rate was similar in both the treatment groups. Thrombocytopenia and anemia are known AEs for ruxolitinib based on its mechanism of action, and BAT involved various therapy options with diverse mechanisms of action with different safety profiles. The efficacy and safety results, and treatment duration of ruxolitinib in the Japanese subgroup were in line with the overall study results.

### Supplementary Information

Below is the link to the electronic supplementary material.

Supplementary file1 (DOCX 18 KB)

## Data Availability

Novartis is committed to sharing with qualified external researchers, access to patient-level data and supporting clinical documents from eligible studies. These requests are reviewed and approved by an independent review panel on the basis of scientific merit. All data provided is anonymized to respect the privacy of patients who have participated in the trial in line with applicable laws and regulations. This trial data availability is according to the criteria and process described on www.clinicalstudydatarequest.com.
