# Supplementary material for: Ruxolitinib in steroid-refractory acute graft-vs-host disease: Japanese subgroup analysis of the randomized REACH2 trial
Source: Int J Hematol. 2024 May 25;120(1):106–16. doi: 10.1007/s12185-024-03772-6 (PMC11226530; doi:10.1007/s12185-024-03772-6)
Supplement: Supplementary file 1 — Supplementary file1 (DOCX 18 KB) [file 12185_2024_3772_MOESM1_ESM.docx]

**Supplementary Tables**

Table S1: Underlying disease history by treatment

| Disease history, n (%) | Ruxolitinib  N = 9 | BAT  N = 21 |
| --- | --- | --- |
| Diagnosis of underlying malignant disease |  |  |
| Acute lymphoblastic leukemia | 1 (11.1) | 2 (9.5) |
| Acute myelogenous leukemia | 5 (55.6) | 5 (23.8) |
| Chronic myelogenous leukemia | 0 | 1 (4.8) |
| Multiple myeloma | 0 | 1 (4.8) |
| Myelodysplastic disorder | 2 (22.2) | 9 (42.9) |
| Non-Hodgkin lymphoma | 0 | 1 (4.8) |
| Other acute leukemia | 1 (11.1) | 0 |
| Other leukemia | 0 | 2 (9.5) |

BAT, best available therapy.

Table S2: Response by organ stage at day 28

| Organ stage (BL vs day 28), number of patients | | | Ruxolitinib  N = 9 | BAT  N = 21 |
| --- | --- | --- | --- | --- |
| Skin | Baseline | Baseline organ involvement^a^ | 4 | 6 |
|  | **Day 28** | Improved | 4 | 5 |
|  |  | Worsened | ─ | 4 |
| Liver | Baseline | Baseline organ involvement^a^ | 0 | 3 |
|  | **Day 28** | Improved | ─ | 1 |
|  |  | Deteriorated | 2 | 4 |
| Upper GI | Baseline | Baseline organ involvement^a^ | 2 | 6 |
|  | **Day 28** | Improved | 2 | 4 |
|  |  | Unchanged | ─ | 2 |
| Lower GI | Baseline | Baseline organ involvement^a^ | 8 | 17 |
|  | **Day 28** | Improved | 7 | 8 |
|  |  | Unchanged | 1 | 5 |
|  |  | Deteriorated | ─ | 3 |

BAT, best available therapy; GI, gastrointestinal.
